# Predicting the cell death responsiveness and sensitization of glioma cells to TRAIL and temozolomide

**DOI:** 10.18632/oncotarget.10973

**Published:** 2016-08-01

**Authors:** Birgit C. Weyhenmeyer, Janis Noonan, Maximilian L. Würstle, Frank A. Lincoln, Grainne Johnston, Markus Rehm, Brona M. Murphy

**Affiliations:** ^1^ Department of Physiology & Medical Physics, Royal College of Surgeons in Ireland, Dublin, Ireland; ^2^ Centre for Systems Medicine, Royal College of Surgeons in Ireland, Dublin, Ireland; ^3^ Institute of Cell Biology and Immunology, Faculty of Energy-, Process- and Biotechnology, University of Stuttgart, Stuttgart, Germany; ^4^ Stuttgart Research Center Systems Biology, University of Stuttgart, Stuttgart, Germany

**Keywords:** glioblastoma, TRAIL, temozolomide, apoptosis, systems biology

## Abstract

Genotoxic chemotherapy with temozolomide (TMZ) is a mainstay of treatment for glioblastoma (GBM); however, at best, TMZ provides only modest survival benefit to a subset of patients. Recent insight into the heterogeneous nature of GBM suggests a more personalized approach to treatment may be necessary to overcome cancer drug resistance and improve patient care. These include novel therapies that can be used both alone and with TMZ to selectively reactivate apoptosis within malignant cells. For this approach to work, reliable molecular signatures that can accurately predict treatment responsiveness need to be identified first. Here, we describe the first proof-of-principle study that merges quantitative protein-based analysis of apoptosis signaling networks with data- and knowledge-driven mathematical systems modeling to predict treatment responsiveness of GBM cell lines to various apoptosis-inducing stimuli. These include monotherapies with TMZ and TRAIL, which activate the intrinsic and extrinsic apoptosis pathways, respectively, as well as combination therapies of TMZ+TRAIL. We also successfully employed this approach to predict whether individual GBM cell lines could be sensitized to TMZ or TRAIL via the selective targeting of Bcl-2/Bcl-xL proteins with ABT-737. Our findings suggest that systems biology-based approaches could assist in personalizing treatment decisions in GBM to optimize cell death induction.

## INTRODUCTION

Glioblastoma (GBM), the most common form of primary brain tumor in humans, is typically aggressive, highly infiltrative, and resistant to conventional therapy. Despite improvements in surgical technique and the addition of temozolomide (TMZ) to the armamentarium, patient median survival remains dismal at 14.6 months, with most experiencing tumor relapse within 7 months of treatment onset [1] and a large proportion gaining no survival advantage to TMZ therapy at all [2, 3]. When successful, this oral alkylating drug induces glioma cell death by causing DNA double strand breaks that eventually lead to growth arrest and activation of cellular apoptosis [4].

To date, the only significant prognostic marker of GBM patient response to TMZ is promoter methylation of the gene encoding for MGMT [5, 6]; specifically, promoter methylation has been identified as a feature of favorable outcome in patients undergoing TMZ therapy for both newly diagnosed GBM and recurrent disease [7]. In the elderly sub-population of patients, *MGMT* testing is now recommended for routine clinical decision making with regards to stratification of therapy; this follows the results of two phase 3 clinical trials that clearly demonstrate ‘unmethylated’ patients benefit more from radiotherapy alone while ‘methylated’ patients benefit more from TMZ chemotherapy alone [8, 9]. In the non-elderly sub-population of GBM patients, however, discordant responses between ‘methylated’ and ‘unmethylated’ sub-groups of patients exist, indicating that the treatment decision to use TMZ in these patients should not be based on this biomarker alone. Nevertheless, *MGMT* testing has become commonplace for patient selection within clinical trials [6] [10–13] and is frequently requested as a prognostic biomarker during patient clinical workup [14].

Irrespective of patient responsiveness to TMZ, the dismal prognosis associated with GBM makes it clear that other therapeutic strategies are required, both as stand-alone treatment options and as sensitizing therapies that can be combined with TMZ to overcome current treatment resistance. In line with this, due to the extremely heterogeneous nature of these tumors [15, 16], it is becoming increasingly evident that such treatment strategies ought to be individualized and tailored to the needs of each GBM patient. Recent efforts in personalizing anti-cancer treatments have focused on therapies that selectively reactivate apoptosis within malignant cells, such as those that promote apoptosis via the Bcl-2 family of regulatory proteins and those that act by binding to death receptors expressed on the surface of the cell.

Tumor necrosis factor-related apoptosis-inducing ligand (TRAIL), the natural ligand for the apoptotic receptors, DR4 and DR5, is one anti-cancer therapy that has been gaining momentum in recent years [17, 18]. Using extrinsic agents like TRAIL has two putative advantages over intrinsic agents: firstly, TRAIL can trigger apoptosis independently of p53, which is commonly mutated in primary (28%) and secondary (65%) GBM patients [19], contributing, in part, to TMZ resistance [20]; and secondly, TRAIL can kill cancer cells without conferring significant toxicity to normal cells [21, 22]. Several TRAIL-based therapies, including the human recombinant TRAIL ligand (dulanermin), which targets both DR4 and DR5, and agonistic antibodies against DR4 (mapatumumab) and DR5 (drozitumab, lexatumumab, tigatuzumab, LBY-135, and conatumumab) have been assessed within clinical trials [17, 23]. Unfortunately, while these agents are reportedly well tolerated in patients, both alone and in combination with standard therapies, only isolated responses have been observed. It should be noted, however, that these trials involved no degree of patient pre-selection and thus may not reflect a true clinical evaluation of TRAIL-based therapies, which might be efficacious but only for a subset of patients.

With regards to GBM, most glioma cells are resistant to TRAIL monotherapy, although several promising combination treatments to overcome this resistance have been described [24–28]. Particularly encouraging *in vitro* and *in vivo* findings come from the combination of TRAIL and TMZ, which evoke concomitant stimulation of the intrinsic and extrinsic apoptotic pathways [29–31]. Used together, these agents should, in theory, enhance both the likelihood of apoptosis induction as well as the strength of the apoptotic signal. TMZ might also play the role of TRAIL ‘sensitizer’, overcoming resistance by up-regulating the expression of death receptors, leading in turn to substantial caspase activation [29, 30]. Other mechanisms of TRAIL and TMZ resistance are shared, such as an up-regulation of anti-apoptotic and down-regulation of pro-apoptotic Bcl-2 proteins and an over-expression of inhibitor of apoptosis (IAP) proteins [23, 32].

Small molecules that antagonize pro-survival Bcl-2 proteins, namely BH3 mimetics, are currently under pre-clinical and clinical evaluation as single agent anti-cancer therapies and as sensitizers to apoptosis-inducing drugs [33, 34]. One of the most advanced and well characterized small molecule inhibitors is the BH3 mimetic, ABT-737, which predominantly binds to Bcl-2 and Bcl-xL to induce or sensitize cells to apoptosis through the intrinsic pathway. As a single agent, ABT-737 has shown anti-tumor activity in GBM cells *in vitro* [35], while in concert with TRAIL, it has demonstrated efficacious results in an *in vivo* model of GBM [27]. Recently, ABT-737 has also been shown to sensitize gliomas cells to TMZ-induced apoptosis [36].

Despite these promising studies, molecular marker signatures that could facilitate reliable predictions on the responsiveness of individual GBM cases to TMZ and TRAIL alone or in combination currently do not exist. Likewise, tools to predict ABT-737-based sensitization of GBM cells to TMZ or TRAIL have not yet been developed. Here, we addressed these problems in a first pre-clinical proof-of-principle study in which we merged quantitative experimental studies with data- and knowledge-driven mathematical system modeling to predict treatment responsiveness of GBM cell lines.

## RESULTS

### Defining and parameterizing functional groups of cell death regulators for knowledge- and data-driven systems modeling

We previously demonstrated that a knowledge- and data-driven mathematical modeling approach is capable of generating reliable predictions of melanoma cell line responsiveness to both TRAIL- and genotoxic drug-induced cell death, outperforming classical statistical procedures [37]. As part of this approach, biological pathway information on linear and non-linear interplay between cell death regulatory proteins is integrated into data obtained from quantitative measurements of these proteins. This is achieved by defining “functional groups (FGs)” in which multiple proteins are related to each other by simple arithmetic rules (sums, products, or ratios of a small number of proteins), multivariate statistical analysis, and the application of pattern recognition algorithms [37–39]. To apply this approach to the TRAIL- and TMZ-based treatments in GBM, the relevant signaling pathways and the respective FGs first needed to be defined.

**Figure 1:**
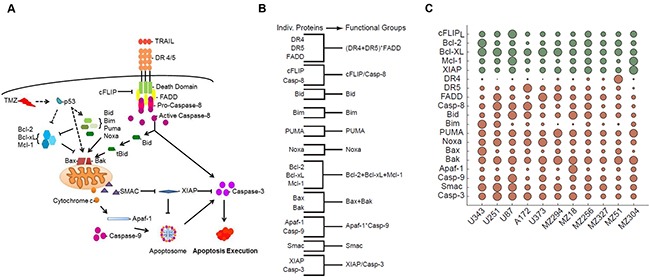
Coding and parameterizing functional groups of apoptosis signaling proteins for knowledge- and data-driven systems modeling **A.** Pathway diagram for intrinsic and extrinsic apoptosis pathways elicited by TMZ and TRAIL, respectively. **B.** Grouping of apoptosis signaling proteins into functional groups by simple arithmetic operations. **C.** Relative levels of protein expression within the GBM cell line panel. Circles summarize 627 quantifications, while circle sizes are proportional to the protein quantities determined from n = 3 independent experiments. Green and red indicate anti- and pro-apoptotic proteins, respectively. Protein expression amounts are provided in Supplementary Table S1. The resulting FG values are provided in Supplementary Table S2.

The apoptosis pathway activated by TRAIL is well characterized and depicted in Figure 1A. Briefly, TRAIL binds to death receptors, DR4 and DR5, on the surface of the cell, resulting in the recruitment of the adaptor protein, Fas-associated death domain (FADD), through death effector domain (DED) interactions. FADD, in turn, recruits pro-caspase-8, pro-caspase-10 and/or cellular-FLIP (cFLIP), thereby forming the multi-protein death-inducing signaling complex (DISC) [40]. Pro-caspase-8 recruitment to the DISC results in its autocatalytic processing to the active caspase-8 enzyme; however, c-FLIP proteins can inhibit this process. If activated, caspase-8 can subsequently go on to activate effector caspases such as caspase-3 to execute the extrinsic apoptotic pathway [41]. Alternatively, caspase-8 can activate the intrinsic pathway via cleavage of the BH3-only protein, Bid. The truncated active form of Bid, tBid, engages with Bax or Bak to induce mitochondrial outer membrane permeabilisation (MOMP) and release of cytochrome *c* and second mitochondria-derived activator of caspases (SMAC) into the cytosol [42]. This mitochondrial pathway of apoptosis, which involves additional BH3-only proteins, such as Bim, Puma and Noxa [43, 44], is also the entry point for cell death signals triggered by chemotherapeutics like TMZ [4, 45–48]. The function of BH3-only proteins and activated Bax or Bak is antagonized by the anti-apoptotic Bcl-2 family proteins, Bcl-2, Bcl-xL and Mcl-1. Once in the cytosol, cytochrome *c* interacts with apoptotic protease activating factor 1 (Apaf-1) to form the heptameric backbone of the apoptosome complex, which in turn recruits and activates caspase-9 [49]. At the same time, SMAC neutralizes the caspase-inhibitory function of X-linked inhibitor of apoptosis protein (XIAP) to assist in the ultimate activation of effector caspases like caspase-3 [50].

We used this knowledge of pathway topology to group the above-mentioned proteins into FGs based on their relationships within the signaling network (Figure 1B). For example, we grouped together Bcl-2, Bcl-xL and Mcl-1 by adding their nM amounts since these represent functionally redundant antagonists of Bax and Bak. Similarly, we grouped together Bax and Bak since they form the mitochondrial pores leading to MOMP. The BH3-only proteins, Bid, Bim, Puma, and Noxa, were kept as separate variables for a number of reasons. Firstly, Bid plays an essential role in driving the activation of the mitochondrial pathway of apoptosis, specifically in response to extrinsic pathway stimulation; therefore, functionally, Bid is largely separate from the other BH3-only proteins. Secondly, the respective relevance and individual importance of Bim, Puma and Noxa in regulating the TMZ-induced intrinsic pathway of apoptosis is still a matter of debate [51]. Furthermore, since these BH3-only proteins may need to be transcriptionally induced or post-translationally modified in response to TMZ, either alone or in combinations, the relevance of their baseline expression levels with regards to determining cell death responsiveness is not yet clear [51–53]. It is also possible that these proteins functionally differ in their capacity to activate/antagonize pro- and anti-apoptotic Bcl-2 family proteins [54, 55]. Thus, keeping these proteins as separate variables allowed us to better understand their respective impact on the model's performance. Of note, in the subsequent results we did not observe a notable improvement in model performance when merging these proteins into one FG (not shown). To define the caspase-9-activating apoptosome complex, we multiplied the protein concentrations of Apaf-1 and caspase-9; this multiplication ensured that the value for this group approached zero when either Apaf-1 or caspase-9 expression was low or absent. We applied the same reasoning to the grouping of DR4, DR5 and FADD; this group represented the DISC. FGs of apoptotic caspases and their antagonists (cFLIP and caspase-8; XIAP and caspase-3) were defined as ratios. SMAC remained as a single protein and accordingly was kept as an individual variable.

To parameterize these 11 FGs with protein data, we carried out quantitative flow cytometry to determine the cell surface expression of death receptors 4 and 5 as well as quantitative immunoblotting to determine the relative protein expression of the other 17 key players of the apoptotic pathways activated by TRAIL and TMZ (Supplementary Table S1). An overview, based on a total of 627 quantifications in 11 cell lines, is provided in Figure 1C. The resulting quantities for the FGs are provided in Supplementary Table S2. The cell lines chosen for this study included both commercially available cell lines, A172, U87, U251, U343, and U373 and a cohort of lines derived from patient GBMs, including both primary (MZ18, MZ51, MZ294, MZ327) and recurrent (MZ256, MZ304) tumors. All of the commercially available lines expressed a methylated *MGMT* promoter whilst among the patient derived cell lines, MZ18, MZ51 and MZ294 expressed a methylated promoter and MZ304, MZ256 and MZ327 expressed an unmethylated promoter. Of note, statistical analysis comparing the relative expression of apoptotic proteins, as listed in Supplementary Table S1, in “methylated” versus “unmethylated” cell lines revealed no relationship between protein expression profiles, methylation status and response to treatment (not shown).

### Functional groups of apoptosis regulators in GBM cell lines can be associated with cell death responsiveness to TMZ and TRAIL monotherapy

**Figure 2:**
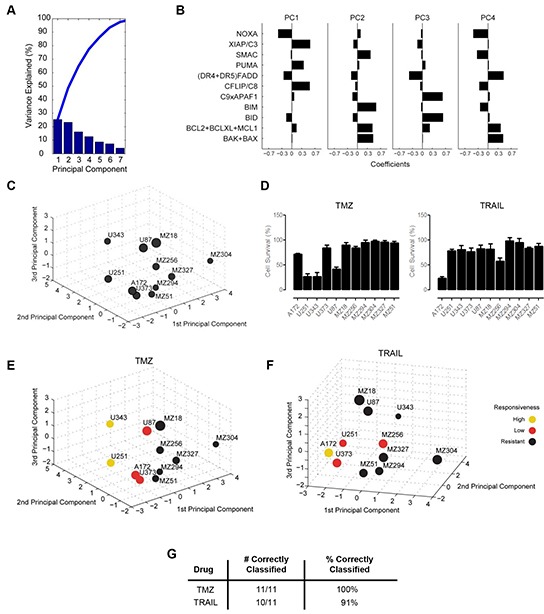
Functional groups of cell death regulators highlight an association between protein expression profiles and cell survival following TMZ and TRAIL treatment **A.** A principal component analysis (PCA) was applied to the functional groups of the GBM cell line panel. Results are shown as a scree plot. Bar graphs show the contribution of each principal component (PC) toward explaining the data variance. **B.** Bar graphs show the coefficients for all functional groups in the first four PCs. Coefficient values are provided in Supplementary Table S3. **C.** Graphical illustration of the distribution of GBM cell lines along the first three PCs. Circle sizes decrease with distance from the observer to aid 3D visualization. **D.** Cell survival following TMZ and TRAIL monotherapy. Cells were treated with TMZ (150 μM) or TRAIL (100 ng/ml) for 96 h followed by cell viability measurement. Data show cell survival relative to control values of 100% (mean ± SEM from n = 3 independent experiments). **E** and **F.** Cell lines in the 3D PC space were color coded according to their responsiveness to TMZ and TRAIL, respectively. **G.** Performance of the response group separation in the 4D PC space by linear discriminant analysis (LDA) is shown by listing the amount of correctly classified cell lines.

Following the parameterization of the FGs, these data were analyzed to determine if any relationship existed between FG values and the responsiveness of each cell line to TMZ or TRAIL. First, we applied a principal component analysis (PCA) to the FG data. This statistical procedure allowed us to accumulate the variance observed in the 11 FGs in the 11 cell lines in a lower number of independent dimensions, referred to as principal components (PCs) (Figure 2A). The variance explained by the respective PCs indicates that the first 4 PCs needed to be retained for the subsequent analyses to capture approximately 70% of the data variance of the original data set, and all subsequent analyses were therefore conducted in this 4D PC space. Each PC is defined by distinct contributions of the different FGs, according to specific weighting coefficients for the FGs (Figure 2B, Supplementary Table S3).

For visualization purposes, the cell-line specific values of the FGs, multiplied with the FG-specific coefficients in the first three 3 PCs, allowed us to position the 11 GBM cell lines within a three dimensional PC space (Figure 2C). The position of the individual cell lines in the PC space is therefore associated with their respective individual protein expression profiles. The distribution of the cell lines within the PC space demonstrated that the cell lines did not form spatially separated clusters, indicating a high degree of heterogeneity between the GBM cell lines (Figure 2C). Additional information on this procedure is provided in the methods section and the published literature [37].

Next, we asked whether the PC space, and therefore the protein composition of the apoptosis signaling network, contains information on whether the cell lines are responsive to TMZ or TRAIL. To this end, we first experimentally measured cell viability following TMZ or TRAIL treatment in all cell lines (Figure 2D). Our data showed that the responsiveness of the cell lines to TMZ or TRAIL varied substantially across the panel and also that individual cell lines frequently responded differently to TMZ or TRAIL. Furthermore, we also noted that very few cell lines responded well to these treatments (Figure 2D). Previous work from our group has highlighted that a reduction in cell survival in TMZ responsive cell lines, U251 and U343, correlates with activation of the intrinsic apoptotic cell death pathway, as demonstrated by a significant increase in the number of condensed nuclei and AnnexinV staining, as well as procaspase-3 activation and PARP cleavage; we also showed that such cell death could be suppressed by caspase inhibition with zVAD-fmk and that no such responses were evident in TMZ-resistant cell lines [56]. To confirm that the TRAIL-sensitive cell lines were undergoing apoptosis via the extrinsic pathway, we examined the processing of procaspase-8 in the TRAIL-sensitive cell line, A172; cleavage of caspase-8 was clearly detected 24 h post TRAIL treatment (Supplementary Figure S1A). We also examined the effect of TRAIL treatment on the executioner caspase, caspase-3, and found that TRAIL treatment also led to the cleavage of pro-caspase-3 after 24 h (Supplementary Figure S1A). Activation of both caspase-8 and caspase-3 was prevented by the combined presence of zVAD-fmk and TRAIL (Supplementary Figure S1A). Furthermore, we detected a significant increase in AnnexinV positive cells following TRAIL treatment (Supplementary Figure S1B), which was also prevented by zVAD-fmk. None of these events were evident in the TRAIL-resistant cell line, MZ304 (Supplementary Figures S1A, S1B).

Subsequently, cell lines were defined treatment-specifically as highly responsive (up to 30% survival), low responsive (30-80% survival) or resistant (>80% survival). According to the definition of response classes, the cell lines positioned in the PC space were color coded (Figure 2E, 2F). Interestingly, it appeared that within the 3D space, resistant cell lines seemed to be spatially separated from cell lines with higher responsiveness (Figure 2E, 2F). While visually this pattern was obvious, we tested whether this separation could likewise be achieved objectively by using linear discriminant analysis (LDA), an algorithm that aims to separate the responsiveness groups by hyperplanes in the 4D PC space. The separation of the response groups by this approach was highly accurate, with all cell lines correctly classified into their respective response groups for TMZ treatment responsiveness (Figure 2G). For TRAIL responsiveness, only one highly responsive cell line was identified, which was pooled with the next highest responsiveness group for all subsequent analyses. LDA correctly classified 10/11 cell lines (91%) for TRAIL responsiveness (Figure 2G). Taken together, these findings indicate that protein data of key cell death regulators together with information on their interplay can be employed to separate GBM cell lines according to their TMZ and TRAIL responsiveness.

### Case specific predictions of TMZ and TRAIL monotherapy responsiveness allow the *in silico* identification of optimal treatments

**Figure 3:**
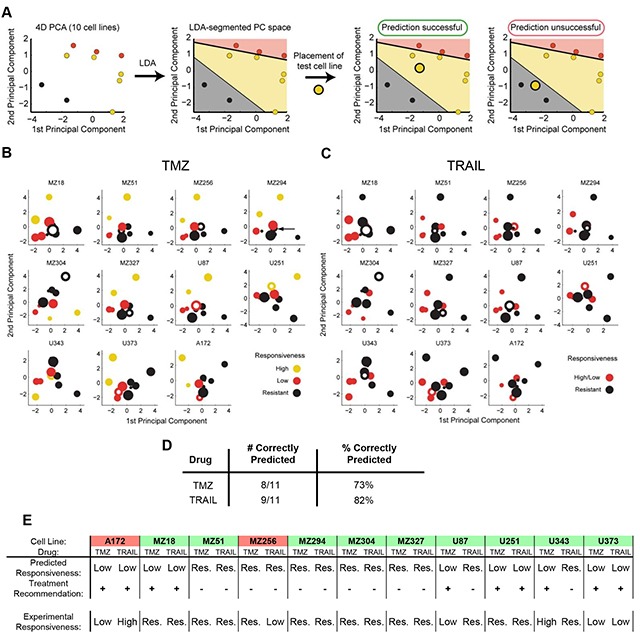
Case-specific predictions of TMZ and TRAIL monotherapy responsiveness in GBM cell lines **A.** Workflow for determining the predictive power of the model using leave-one-out cross-validation (LOOCV). All predictions were performed in 4D PC space. **B, C.** 2D projections of the PC spaces calculated from combinations of 10 cell lines are shown for TMZ and TRAIL treatments. Circle sizes decrease with distance from the viewer, thereby providing information on the third PC dimension. Open circles represent the test cell lines, which were placed into the PC spaces according to their functional group values. **D.** Performance scores for correctly predicting cell line responses to TMZ and TRAIL monotherapy. **E.** Case-specific predictions on TMZ and TRAIL responsiveness allow the *in silico* identification of optimal treatment options. Treatment recommendations were made according to the highest predicted responsiveness to TMZ or TRAIL and validated against experimental data. Green background indicates cases where optimal treatment options could be identified. Red background indicates cases where the better treatment option could be missed.

We next investigated whether the above findings can assist in predicting the responsiveness of individual cell lines to TMZ or TRAIL. We hypothesized that conducting the above analyses with a reduced number of cell lines may be sufficient to generate a PC space that can be segmented into spatial regions that reflect different levels of treatment responsiveness. We conducted a leave-one-out cross validation (LOOCV) in which we used the data from 10 cell lines and subsequently positioned the 11^th^ cell line into the LDA segmented PC space. If the placed cell line (test cell line) was positioned within the correct response region, the prediction was considered to be correct (Figure 3A). A visualization of the results limited to the first two PCs for TMZ and TRAIL response predictions is shown in Figure 3B, 3C. The prediction accuracies are summarized in Figure 3D; this demonstrates that TMZ and TRAIL responsiveness can be predicted with high accuracy (73% and 82% correct predictions, respectively).

In situations where multiple treatment options are available, treatment decision tools are required to assist in pre-selecting the optimal treatment option. An optimal treatment in our case (TMZ vs. TRAIL) was defined as the treatment that induces higher amounts of cell death for a given cell line. If both drugs cause similar amounts of cell death, either treatment suggestion was considered acceptable. Predictions were considered to have failed when the model recommended the treatment option with a lower effect on cell viability than the alternative treatment option. The performance of the model as a treatment decision tool is shown in Figure 3E. Treatment recommendations for the 11 cell lines were correct in 82% (9/11) of cases. These proof-of-concept results demonstrate that our modeling approach has the potential to identify best treatment options for individual cell lines with high accuracy.

### Capability to predict synergistic responses to TMZ and TRAIL

It has previously been reported that combination therapy with TMZ/genotoxic agents and TRAIL may significantly enhance the responsiveness of highly treatment resistant cancer models, such as GBM cell lines [29–31]. We therefore next studied the responsiveness of the cell line panel to TMZ/TRAIL combination treatment. First, we investigated whether the response groups could be separated in the PC space and then we determined whether overall responsiveness as well as response synergies in these cell lines could be reliably predicted.

**Figure 4:**
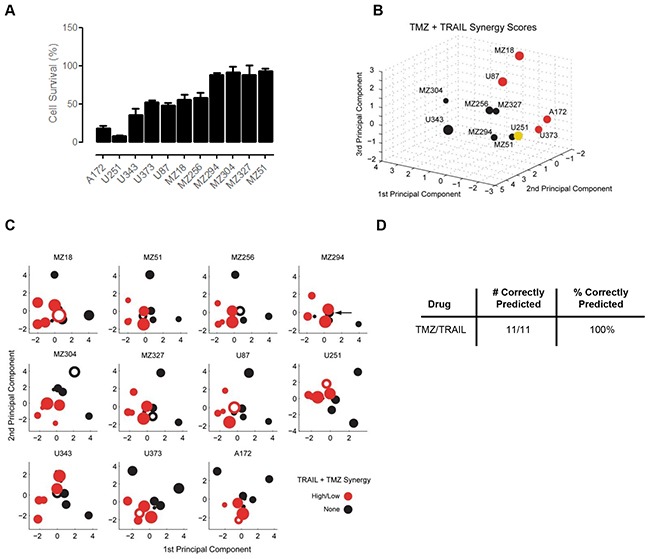
Predicting the synergy of TRAIL and TMZ combined therapy **A.** Cell survival following TMZ and TRAIL combined therapy. Cells were treated with TMZ (150 μM) and TRAIL (100 ng/ml) for 96 h and MTT assays were performed to assess cell viability. Data show cell survival relative to control values of 100% (mean ± SEM from n = 3 independent experiments). **B.** Cell lines in the 3D space were separated according to their Combination Index (CI) Value and color coded according to their response synergies to TMZ and TRAIL combined treatment. Visual inspection indicates the response regions are spatially separated. **C.** 2D projections of the PC spaces calculated from combinations of 10 cell lines are shown for TMZ/TRAIL combined treatments. Circle sizes decrease with distance from the viewer, thereby providing information on the third PC dimension. Open circles represent the test cell lines, which were placed into the PC spaces according to their CI Value. Note that the U251 cell line had to be grouped with the low responding cell lines in this analysis since it was alone in its response group. **D.** Performance scores for correctly predicting synergistic responses to TMZ/TRAIL combination therapy.

Overall, the responsiveness of the GBM cell line panel to TMZ/TRAIL treatment was improved when compared to the single agent treatments (Figure 4A, Figure 2D); this reduction in cell viability (Figure 4A) correlated with an increase in apoptotic cell death in TMZ and TRAIL-treated U251 cells, as evidenced by PARP cleavage and the prevention of such cleavage upon caspase inhibition (Supplementary Figure S1C), a significant increase in the number of Hoechst-labelled condensed nuclei (Supplementary Figure S1D), and caspase-3 substrate cleavage (Supplementary Figure S1E). No such cell death-related changes were evident in the treatment-resistant cell line, MZ294 (Supplementary Figure S1C, S1D, S1E). As with the single agent treatments, the response pattern to TMZ/TRAIL treatment was heterogeneous across the cell line panel, with both resistant and responsive cell lines identified (Figure 4A). Color coding the cell lines according to TMZ+TRAIL responsiveness again indicated that regions in the PC space appear to be associated with different levels of treatment responsiveness, and that these spatial regions can be separated by LDA (Supplementary Figure S2A, S1B). To test the predictive capacity of the model for TMZ+TRAIL combination treatment, we again conducted LOOCV (Supplementary Figure S2C). However, the responsiveness of only 6 out of the 11 cell lines was predicted correctly (55%) (Supplementary Figure S2D).

Since the capacity of the model to predict cell line responsiveness to the dual treatment strategy of TMZ and TRAIL was limited, we next considered whether our modeling approach was better suited to study and predict response synergies. To this end, we first calculated the (CI) Value of each cell line using Webb's fractional product method [56], which provided us with a ‘synergy score’ for the combination treatment for each cell line (Supplementary Table S4); this was then used to place the cell lines in the PC space. Using this approach, we obtained regions in the PC space that allowed us to separate the cell lines by response synergies (Figure 4B). Furthermore, once we performed LOOCV using the CI Values, we found that we could successfully predict response synergies to TMZ and TRAIL combination therapy with 100% accuracy (Figure 4C, 4D). Taken together, these results indicate that while prediction accuracies for the overall responsiveness to TMZ and TRAIL were limited, apoptosis protein expression patterns together with our modeling approach, were particularly powerful in predicting response synergies.

### Accurate prediction of TRAIL sensitization by Bcl-2/Bcl-xL antagonist ABT-737

The above analyses demonstrated that it is possible to predict from baseline protein expression profiles whether individual cell lines will respond to TMZ or TRAIL monotherapy. Since many of the GBM cell lines were resistant to TMZ or TRAIL or only responded poorly, we next investigated if our systems modeling approach could be used to predict whether poorly responsive cell lines could be sensitized to treatment by ABT-737. ABT-737 specifically antagonizes Bcl-2 and Bcl-xL, the major anti-apoptotic members of the Bcl-2 protein family, by binding to a hydrophobic groove of these proteins, thereby displacing bound activator BH3-only proteins and enabling the activation of pro-apoptotic Bax and Bak [57].

**Figure 5:**
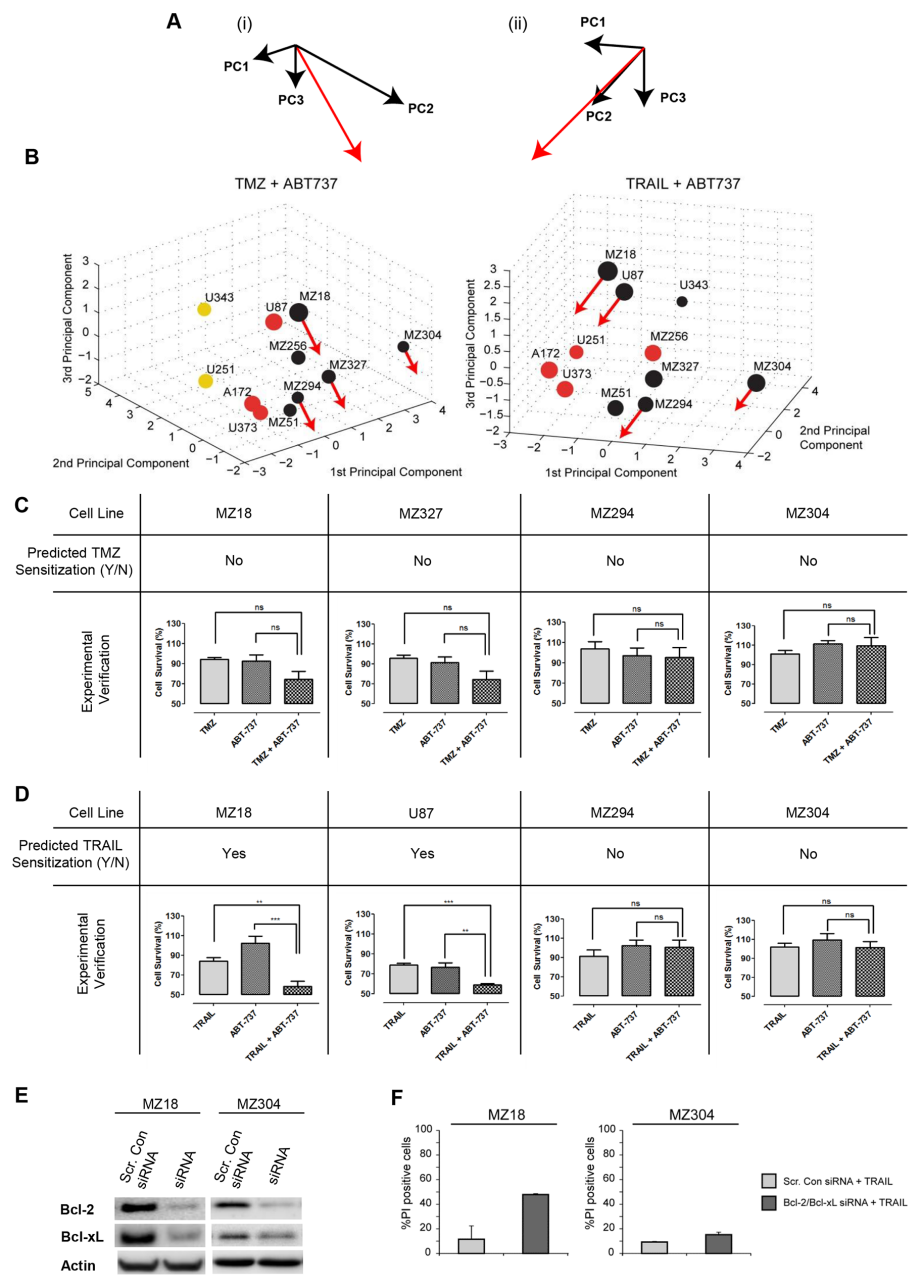
Prediction and experimental validation of GBM cell line sensitization to TRAIL or TMZ treatment by ABT-737 **A.** A movement vector (red) was calculated by combining the weightings for the (Bcl-2+Bcl-xL+Mcl-1) functional group in the first 3 PCs. (i) and (ii) provide different perspectives on the vectors, compatible with 3D PC spaces for TMZ or TRAIL responsiveness. **B.** 3D PC spaces for TMZ and TRAIL responsiveness with movement vectors applied to test cell lines. The length of the movement vectors was calculated from the respective Bcl-2 and Bcl-xL content of the cell lines. Movement of treatment resistant cell lines towards regions populated by cell lines with higher treatment responsiveness predicts sensitization by the Bcl-2/Bcl-xL antagonist ABT-737. **C, D, E.** Experimental validation of predictions. Cell viability was measured for single and combination treatments. (C) Cells were treated with TMZ (150 μM) ± ABT-737 (1 μM) for 48 h. (D) Cells were treated with TRAIL (100 ng/ml) ± ABT-737 (1 μM) for 48 h. Data show cell viability (mean ± SEM from n = 3 independent experiments) in relation to untreated controls (100%). One-way ANOVA with Bonferroni comparisons was performed for statistical analysis. **p<0.01, ***p<0.001. ns = not significant. (E) Western blot analysis to confirm the successful knockdown of Bcl-2 and Bcl-xL. MZ18 and MZ304 cells were transfected with Bcl-2 and Bcl-xL esiRNA. **F.** siRNA depletion of Bcl-2 and Bcl-xL strongly sensitizes MZ18 but not MZ304 cells to TRAIL. Data show the number of PI^+^ cells (mean ± S.D. from triplicate samples) following treatment with TRAIL (100 ng/ml). Experiments were repeated with similar results.

To generate predictions on which cell lines could be sensitized to TMZ or TRAIL by ABT-737, we first determined how the position of such cell lines in the PC space would change upon elimination of Bcl-2 and Bcl-xL. This repositioning was determined from the PCA results and the cell lines' protein profiles. The vector for the movement direction (Figure 5A) was determined by inverting and combining the coefficients of the FG for the anti-apoptotic Bcl-2 proteins (Figure 2B); the distance moved depended on the amount of Bcl-2 and Bcl-xL that contributed to the (Bcl-2+Bcl-xL+Mcl-1) FG value in the respective cell lines (high amounts of Bcl-2 and Bcl-xL caused a more pronounced displacement). The resulting repositioning vectors were then applied to four representative cell lines that poorly responded to TMZ or TRAIL (MZ18, MZ294, MZ304, MZ327 cell lines and MZ18, U87, MZ304, MZ294 cell lines, respectively). For TMZ + ABT737 treatments, the movement vectors indicated that none of the cell lines would reposition closer to responding cell lines (Figure 5B), suggesting that ABT-737 co-treatment would not sensitize these glioma cells to TMZ. For TRAIL + ABT737 treatments, the movement vectors indicated that MZ18 and U87, but not MZ294 and MZ304, cell lines would reposition closer to responding cell lines and thus be sensitized to TRAIL (Figure 5B).

These predictions were then validated experimentally. The selected cell lines were treated with either TMZ or TRAIL in the presence and absence of ABT-737 (1 μM) for 48 h. Cell viability measurements demonstrated that ABT-737 selectively sensitized MZ18 and U87 cells to TRAIL treatment, whereas ABT-737 did not sensitize any of the other cell lines to TMZ or TRAIL (Figure 5C, 5D). ABT-737 did not reduce cell viability on its own. These findings demonstrate that all predictions regarding ABT-737-based sensitization could be validated (8/8), with our approach able to identify the subset of GBM cell lines that could be successfully sensitized to TRAIL by ABT-737 co-treatment. To further validate these predictions, we used siRNA to knockdown Bcl-2 and Bcl-xL in MZ18 and MZ304 cells. As expected, our findings demonstrate that successful knockdown of Bcl-2 and Bcl-xL is sufficient to strongly sensitize MZ18 cells to TRAIL monotherapy. Our findings also demonstrate that, as predicted, Bcl-2 and Bcl-xL knockdown failed to notably raise the sensitivity of MZ304 cells to TRAIL monotherapy (<20% cell death). (Figure 5E, 5F). Overall, these findings demonstrate that the sensitizing effect of pharmacologically neutralizing anti-apoptotic Bcl-2 family members or by siRNA-based depletion can be accurately predicted by our approach.

## DISCUSSION

Efficient induction of cell death is the aim of many anti-cancer therapies; thus, it follows that a cellular defect in cell death signaling pathways like apoptosis underlies one way in which these therapies can fail [32]. In apoptosis, death decisions are tightly controlled through multi-factorial and non-linear interplay of numerous regulatory proteins [58]; for this reason, in-depth molecular analysis of any single protein, whether it be death-inducing or survival-promoting, is unlikely to yield an accurate prediction of drug responsiveness. Indeed, it is worth noting that no individual cell death regulating protein has so far been identified as a predictive or prognostic biomarker for GBM [59]. Instead, due to the complexity of apoptosis signaling, it seems rational that any potential insight gained from this cell death pathway should require analysis of all the key players involved. This, coupled with knowledge of how these players interact, has the potential to provide us with a much greater overview of a cell's capability to undergo apoptosis when confronted with a therapy that ultimately aims to achieve this mode of cell death. In this study, we applied this theory to both intrinsic (TMZ) and extrinsic (TRAIL) apoptosis-inducing therapies and successfully separated our panel of GBM cell lines according to their treatment response with 100% and 91% accuracy, respectively (Figure 2G). This indicates that the complexity of the apoptosis signaling network as a whole needs to be taken into account for the implementation of successful predictive and prognostic strategies in GBM.

The functional groups defined in our study represent low numbers of proteins logically inter-related according to their biological interplay. This approach towards systems modeling is notably simplistic when compared to alternative modeling strategies, such as deterministic mathematical models that build on the use of differential equations [60]. The modeling strategy chosen here has the distinct advantage of being more versatile and easy to implement, amend and extend going forward. For example, to build on our proof-of-concept study with subsequent translational studies, we could include additional independent variables that pertain, for example, to the sub-type of GBM, thereby gaining knowledge of the driver mutations and their effect on cell death/survival signals. Age, Karnofsky performance score, extent of surgical resection and other clinicopathological factors influencing prognosis could also be included [61].

Importantly, once our cell lines were distributed in the PC space according to their protein expression profiles, we found little evidence of cell line overlap or clustering, indicative of the widespread heterogeneity found between the FGs of each cell line. This mirrors the extensive molecular heterogeneity found between GBM patients [15, 16] and backs the theory that a ‘one-size-fits-all’ approach to treating this disease, particularly with an apoptosis-targeted therapy, may not be valid [62]. Instead, in line with advocates of personalized medicine, it is conceivable that patients could be treated based on their unique set of molecular alterations in, or expression of, apoptosis regulators. For this, treatment decision tools such as ours will need to be developed and clinically validated in the future to assist in the pre-selection and stratification of patients for optimal therapies.

The prediction accuracy of our model was 73% and 82% for TMZ and TRAIL treatments, respectively. The higher performance score for TRAIL was likely attributable to the fact that we included all critical TRAIL signaling components in our model. Furthermore, TRAIL-induced apoptosis is capable of proceeding independently of protein neo-synthesis, which suggests that baseline expression values for TRAIL signaling proteins is sufficient to predict the capacity of a cell to undergo TRAIL-induced apoptosis. The one obvious omission to the model, which may resolve any prediction error, is quantitative input for the TRAIL decoy receptors (DcR1 and DcR2). However, the level of decoy receptor expression in glioma cells is reportedly very low and therefore unlikely to account for TRAIL resistance [63]. Of course, we can't exclude the possibility that other cellular events could be influencing TRAIL susceptibility, such as survival signaling [64] and induction of cytoprotective autophagy [65, 66]. For example, it is now known that in some cellular contexts TRAIL can engage NF_k_B, PKB/Akt and MAPKs signaling pathways, thereby contributing to cancer cell proliferation and migration as well as resistance to TRAIL-induced apoptosis [67]. Improving the predictive capacity for TMZ from baseline protein expression profiles may prove to be more difficult given that apoptosis occurs as a consequence of DNA damage and requires transcriptional and translational responses [4]. However, in this regard, future model extensions could take into account additional upstream events, such as DNA repair activity and p53 status as a main driver of transcriptional BH3-only protein induction [68, 69].

Cell line responsiveness to TMZ and TRAIL varied substantially, with individual cell lines frequently responding differently to TMZ and TRAIL monotherapy. This gave us the opportunity to assess whether our model could be exploited as a treatment decision tool. The prediction accuracy of our model was 82%, with treatment recommendations correct 9 times out of 11. However, it should be noted that few cell lines responded well to either treatment, highlighting once again the profound resistance of glioma cells to apoptotic stimuli and the need for more effective and rational combination therapies. In recent years, there has been an abundance of studies reporting on mechanisms to overcome apoptosis resistance in GBM for both established and novel therapies [24–32]. For the purpose of this study, we first focused on TMZ and TRAIL combination therapy, which in theory has the propensity to activate both the intrinsic and extrinsic apoptotic pathways, thereby enhancing both the likelihood of apoptosis induction as well as the strength of the apoptotic signal. In this case, our model only predicted the responsiveness of cell lines to TMZ+TRAIL with 55% accuracy (Supplementary Figure S2D). This indicates that other factors need to be taken into account to more accurately predict TMZ+TRAIL responsiveness. Such factors may include additional molecular information, such as genetic alterations, resulting in the inactivation of apoptosis proteins or that may affect death/survival signals. Similarly, additional pathway cross-talk and/or the potentiation of signaling processes may need to be taken into account. For example, to address the synergistic manner in which TMZ and TRAIL come together to enhance apoptosis and in an attempt to determine whether knowledge of this synergy improves the predictive power of the model in co-treatment scenarios, we calculated the CI Value of each cell line [57]; this in turn allowed us to predict with 100% accuracy the response synergies of individual cells lines to TMZ+TRAIL (Figure 4C, 4D).

Strikingly, we were also able to predict cell line sensitivity to TMZ and TRAIL upon their co-treatment with ABT-737 with 100% accuracy, highlighting that the consequences of a targeted intervention are captured by our approach; this finding was further validated by siRNA depletion of Bcl-2 and Bcl-xL. In light of the recent FDA approval of the first Bcl-2 inhibitor, Venetoclax (ABT-199), for patients with chronic lymphocytic leukemia with the 17p deletion, as well as the encouraging phase I-III clinical trials in development for Venetoclax as a monotherapy and/or combination therapy for various types of cancer [70], our capability to accurately predict the treatment outcome of a targeted intervention within the apoptosis signaling cascade is a particularly pertinent finding. Interestingly, ABT-737 failed to sensitize any GBM cell line to TMZ therapy, whilst in contrast MZ18 and U87 cells were sensitized to TRAIL. On the one hand, this may suggest that the expression levels of Bcl-2 and Bcl-xL are irrelevant to TMZ resistance, at least within the expression ranges observed in our cell line panel, but on the other hand it also highlights the potential superiority of TRAIL-based therapies. TRAIL has long been perceived as a potential attractive chemotherapeutic agent, since it can kill cancer cells without conferring significant toxicity to normal cells [21], however, to date, no TRAIL-based therapy has demonstrated both potency and lack of systemic toxicity in any clinical trial [17, 22, 23]. Besides the limitations of previous TRAIL-based therapeutics, that are now being addressed in the design of superior 2^nd^ generation receptor ligands [71, 72], it is our conjecture that the lack of patient pre-selection in these trials may have contributed to their disappointing outcomes.

With the era of personalized medicine looming, our approach, if validated translationally in subsequent studies, could help to stratify patients for TRAIL mono- or combination therapies. Such translational studies would initially require use of large-scale quantitative protein data from fresh or archived GBM tissue as well as the identification of high quality antibodies for the use of high through-put screening methods, like reverse phase protein or tissue micro arrays. For a clinical setting, our model could be refined to focus on cancer stem cell populations, believed to be the tumor initiating cells that drive the growth, progression and invasion of GBMs as well as their post-treatment recurrence [73–75]. In this regard, quantitative protein analysis of this highly tumorigenic sub-population of cells could provide us with crucial insight into whether tumor cells not surgically removed will respond to treatment.

In conclusion, we have described the first pre-clinical proof-of-principle study that merges quantitative protein-based analysis of apoptosis signaling networks with data- and knowledge-driven mathematical systems modeling to predict treatment responsiveness of GBM cell lines to various apoptosis-inducing stimuli. Furthermore, we have shown that a systems biology-based approach could assist in personalizing treatment decisions in GBM to optimize cell death induction.

## MATERIALS AND METHODS

### Materials

TMZ was purchased from Sigma-Aldrich Ireland Ltd (Dublin, Ireland). Human recombinant TRAIL was purchased from Enzo Life Science (UK) Ltd (Exeter, United Kingdom). ABT-737 was purchased from Biorbyt (Riverside, United Kingdom), and Bcl-2 and Bcl-xL esiRNAs were purchased from Sigma-Aldrich Ireland Ltd (Dublin, Ireland).

### GBM cell lines

GBM cell lines A172, U87, U251, U343 and U373 are commercially available from the ATCC. GBM cell lines MZ18, MZ51, MZ294, MZ304, MZ327, and MZ256 were kindly donated by Professor Donat Kögel of Johann Wolfgang Goethe University Hospital, Frankfurt, Germany and have previously been characterized [76]. The cells were grown in Dulbecco's modified Eagle's medium (DMEM) with 10% heat-inactivated fetal calf serum, 100 U/ml penicillin and 100 mg/ml streptomycin and were maintained in a humidified incubator at 37°C and 5% CO_2._

### Western blot analysis

In order to determine the basal expression level of apoptotic proteins, GBM cell lines were homogenized in lysis buffer containing 0.5 mmol/l Tris-HCl (pH6.8), 2% SDS (w/v), 10% glycerine (w/v), and protease and phosphatase inhibitor cocktails (Sigma-Aldrich). After determining the protein concentration of samples using a BCA protein assay (Pierce, Rockford, IL, USA), 20 μg samples were boiled in gel-loading buffer and separated on 10-15% SDS-PAGE gels. Proteins were transferred to nitrocellulose membranes using the iBlot gel transfer device (Life Technologies, Invitrogen, Paisley, Scotland). The membranes were blocked in 5% non-fat milk in TBST for 1 h at room temperature prior to being incubated with primary antibodies overnight at 4°C. The following primary antibodies were used: rabbit polyclonal Apaf-1 (Cat# 16941, Millipore); rabbit polyclonal Bak (Cat# sc-832, Santa Cruz); rabbit polyclonal Bax (Cat# 06-499, Upstate Biotechnology); mouse monoclonal Bcl-2 (Cat# sc-509, Santa Cruz); mouse monoclonal Bcl-xL (Cat# sc-8392); rabbit polyclonal Bid (Cat# 2002, Cell Signaling); rabbit monoclonal Bim (Cat# 2933, Cell Signaling); rabbit polyclonal caspase-3 (Cat# 9662, Cell Signaling); mouse monoclonal caspase-8 (Cat# ALX-804-242, Enzo Life Sciences); rabbit polyclonal caspase-9 (Cat# 9502, Cell Signaling); mouse monoclonal cFLIP (Cat# ALX-804-428, Enzo Life Sciences); mouse monoclonal FADD (Cat# 610399, BD Biosciences); mouse monoclonal Mcl-1 (Cat# 559027, BD Biosciences); mouse monoclonal Noxa (Cat# 13654, Abcam); rabbit polyclonal PUMA (Cat# 3041, ProSci Incorporated); mouse monoclonal SMAC/DIABLO (Cat# 2954, Cell Signaling); and mouse monoclonal XIAP (Cat# 610763, BD Biosciences); and mouse monoclonal β-actin (Cat# A5441, Sigma-Aldrich). Membranes were then washed three times with TBST for 5 min prior to being incubated with peroxidase-conjugated secondary antibodies (anti-mouse or anti-rabbit; Millipore) for 1 h at room temperature. Protein bands were visualized using the Immobilin western chemiluminescence HRP substrate (Millipore) and images were captured using a LAS-4000 imager equipped with a cooled 12 bit digital CCD camera (Fujifilm UK Ltd, Bedfordshire, UK). To guarantee accurate quantifications, special care was taken not to over-expose the protein bands. Densitometry was carried out on 12-bit raw images using ImageJ 1.4.10 software (National Institute of Heath, Bethesda, MD, USA; http://rsb.info.nih.gov/ij). For each protein, the integrated density of the signal was measured, corrected for background signals and normalized to a β-actin loading control. Standard curves from HeLa cell extracts (5-20 mg) were run concurrently with the GBM cell line extracts to ensure linearity of the signal detection range. The absolute concentration of most proteins of interest was previously determined in HeLa cells [77–79], so that HeLa cell signals could be used to calculate protein concentrations in GBM cell lines. Quantifications were carried out on at least 3 independent membranes. Cleavage of caspase-8 and caspase-3 was also assessed in A172 and MZ304 cells following TRAIL treatment (100 ng/ml) in the presence of the caspase inhibitor, zVAD-fmk (150 μM; 30 min pre-treatment) for 24 and 96 h, respectively. Cleavage of PARP was assessed in MZ294 and U251 cells following the combined treatment of TRAIL (100 ng/ml) + TMZ (150 μM) in the presence or absence of zVAD-fmk (150 μM; 30 min pre-treatment) for 96 h.

### Flow cytometry

The basal surface expression of DR4 and DR5 were assessed using a BD LSR II flow cytometer (BD Biosciences). Briefly, 1 × 10^5^ cells were seeded into 6 well plates and allowed to adhere overnight. Cells were then harvested with trypsin-EDTA and pelleted by centrifugation at 1200 rpm for 5 min at 4°C. Following incubation with a blocking buffer (0.5% BSA) on ice for 20 min, cells were incubated with a mouse monoclonal antibody for DR4 (Cat# ab13890, Abcam; 1:100 dilution) or DR5 (Cat# ALX-804-914-0100, Enzo life sciences; 1:100 dilution) for 30 min, pelleted and washed x 3 with PBS, and then incubated with a secondary anti-mouse FITC-conjugated antibody (Life Technologies, Invitrogen, Paisley, Scotland; 1:200 dilution) for 30 min in the dark. Controls were stained with secondary antibody only. FITC was excited at 488 nm and fluorescence emission was collected in the FL1 channel through a 520 nm band-pass filter. A total of 1 × 10^4^ gated cells were acquired for each cell line. The relative expression of DR4 and DR5 was determined by comparison of specific staining intensities compared to individual cell line negative controls. For cell death measurements, flow cytometry was used to assess the number of AnnexinV^+^ cells following treatment with TRAIL (100 ng/ml) or TRAIL+TMZ (150 μM) in the presence or absence of zVAD-fmk (150 μM; 30 min pre-treatment) for 96 h as well as the number of PI^+^ cells following transfection with Bcl-2/Bcl-xL siRNA and treatment with TRAIL (100 ng/ml) for 48 h. Briefly, cells were seeded into 6-well plates and allowed to adhere overnight. Following appropriate treatment conditions, cells were pelleted and incubated in 100μl of binding buffer (10 nM HEPES, 135 nM NaCl, 5 mM CaCl_2_) containing AnnexinV- FITC conjugate (5 μl/ml) (BioVision, Moutain View, CA, USA) for 10 min on ice in the dark or in DMEM containing PI (1.33 μg/ml) for 15 min at 37°C. FITC was excited at 488 nm and fluorescence emission was collected in the FL1 channel through a 520 nm band-pass filter. PI was excited at 561 nm and fluorescence emission was collected through a 605/40 nm band-pass filter and a 570 nm long pass filter. A total of 1 × 10^4^ gated cells were acquired for each cell line.

### Cell transfection

To downregulate Bcl-2 and Bcl-xL protein expression, we used endoribonuclease-prepared small interfering RNAs (esiRNAs), which are comprised of a heterogeneous mixture of siRNAs that all target the same mRNA sequence (Cat# EHU135281 and EHU087041, Sigma-Aldrich). Briefly, MZ18 and MZ304 cells were transfected with 1 μg of Bcl-2 esiRNA and 1 μg of Bcl-xL esiRNA for 4 h using LipofectamineTM 2000 in antibiotic-free Opti-MEM medium (Life Technologies, Invitrogen, Paisley, Scotland). Following this incubation period, the transfection mixture was replaced with fresh DMEM and the cells were allowed to recover for 48 h, after which they were used for experiments.

### Cell viability measurements

GBM cell lines were seeded into 96-well plates (2000 cells/well) and allowed to adhere overnight. To validate cell line responsiveness to TMZ and TRAIL monotherapy, cells were treated at clinically-informed “maximum effect” (Emax) conditions and accordingly were treated with TMZ (150 μM) or TRAIL (100 ng/ml) for 96 h. To confirm cell lines responsiveness to TRAIL and TMZ combined therapy, cells were treated with TRAIL (100 ng/ml) and TMZ (150 μM) for 96 h. To substantiate the sensitization of cell lines to TRAIL or TMZ with ABT-737, cells were either treated with TRAIL (100 ng/ml) in the presence or absence of ABT-737 (1 μM) for 48 h or with TMZ (150 μM) in the presence or absence of ABT-737 (1 μM) for 48 h. Following treatment, cells were incubated with thiazolyl blue tetrazolium bromide (MTT; 5mg/ml; Sigma-Aldrich Ireland Ltd) for 4 h at 37°C, after which the medium was aspirated and replaced with DMSO (200 μl). The absorbance of each sample was measured at 560 nm using a microplate reader (GENios, Tecan, Mannedorf, Switzerland). MTT is a yellow tetrazole that is converted to dark blue/purple formazan by mitochondrial dehydrogenase of living cells. Thus, the absorbance value for each sample was considered proportional to the number of viable cells.

### Hoechst staining

MZ294 and U251 cells were stained with Hoechst 33258 (1 μg/ml) for 20 min. Changes in nuclear morphology were detected by epifluorescence microscopy (Nikon Eclipse TE300). Normal bright field images were taken to examine morphological changes, such as cell shrinkage and membrane blebbing.

### Caspase-3 substrate cleavage

DEVDase (Caspase-3-like) activity was determined fluorometrically using carbobenzoxy-Asp-Glu-Val-Asp-7-amino-4-methyl-coumarin (DEVD-AMC) as substrate. Cleavage of DEVD-AMC to liberate free AMC was monitored by measuring fluorescence after 1 and 2 h intervals. Protein content was determined using Pierce Coomassie Plus Protein assay reagent (Perbio, Northumberland, UK). Caspase activity is expressed as a change in fluorescent units per hour and per microgram protein.

### Statistical analysis

Student's t-test or one-way ANOVA with post hoc Bonferroni test were performed to identify statistically significant differences between treatment groups. *P*-values are indicated in the figure panels and legends.

### Data processing and analysis for knowledge- and data-driven systems modeling

All data processing and analysis was performed using a programming code developed for MATLAB 2007b (The Mathworks, UK), equipped with the statistics toolbox; a detailed description of the procedures has previously been published [37]. In brief, following the integration of protein expression data into functional groups, a PCA was performed [38, 39]. PCs with eigenvalues above 1 were retained, according to the Kaiser criterion [80, 81]. Scatter plots were generated from the first three PCs to visualize the data. LDA [82] was employed to determine the accuracy of response class separations in the PC space. LOOCV was applied to iteratively test the prediction capacity of the model for the entire cell line ensemble. LDA was applied after each iteration to determine if the test cell lines positioned in the PC space region that corresponded to its drug responsiveness. To predict sensitization effects of ABT-737 in co-treatment scenarios, the protein values for the target proteins, Bcl-2 and Bcl-xL, were set to zero and the re-positioning of cell lines of choice in the PC space was investigated. Re-positioning towards PC space regions of higher responsiveness were interpreted as a prediction for successful sensitization. A detailed description for this procedure is available in [37].

## SUPPLEMENTARY FIGURES AND TABLES





## Abbreviations

TMZ: temozolomide
GBM: glioblastoma
TRAIL: tumor necrosis factor-related apoptosis-inducing ligand
DR4/5: death receptor 4/5
IAP: inhibitor of apoptosis protein
FG: functional groups
FADD: fas-associated death domain
DISC: death-inducing signaling complex
DED: death effector domain
MOMP: mitochondrial outer membrane permeabilisation
SMAC: second mitochondria-derived activator of caspases
Apaf-1: apoptotic protease activating factor 1
PCA: principle component analysis
LDA: linear discriminant analysis
LOOCV: leave-one-out cross validation
EGFR: epidermal growth factor receptor
PDGFR: platelet-derived growth factor receptor
siRNA: small interfering RNA
