# Supplementary material for: Predicting the cell death responsiveness and sensitization of glioma cells to TRAIL and temozolomide
Source: Oncotarget. 2016 Aug 1;7(38):61295–311. doi: 10.18632/oncotarget.10973 (PMC5308652; doi:10.18632/oncotarget.10973)
Supplement: Supplementary file 1 [file oncotarget-07-61295-s001.pdf]

Predicting the cell death responsiveness and sensitization of glioma cells to TRAIL and temozolomide

SUPPLEMENTARY FIGURES AND TABLES

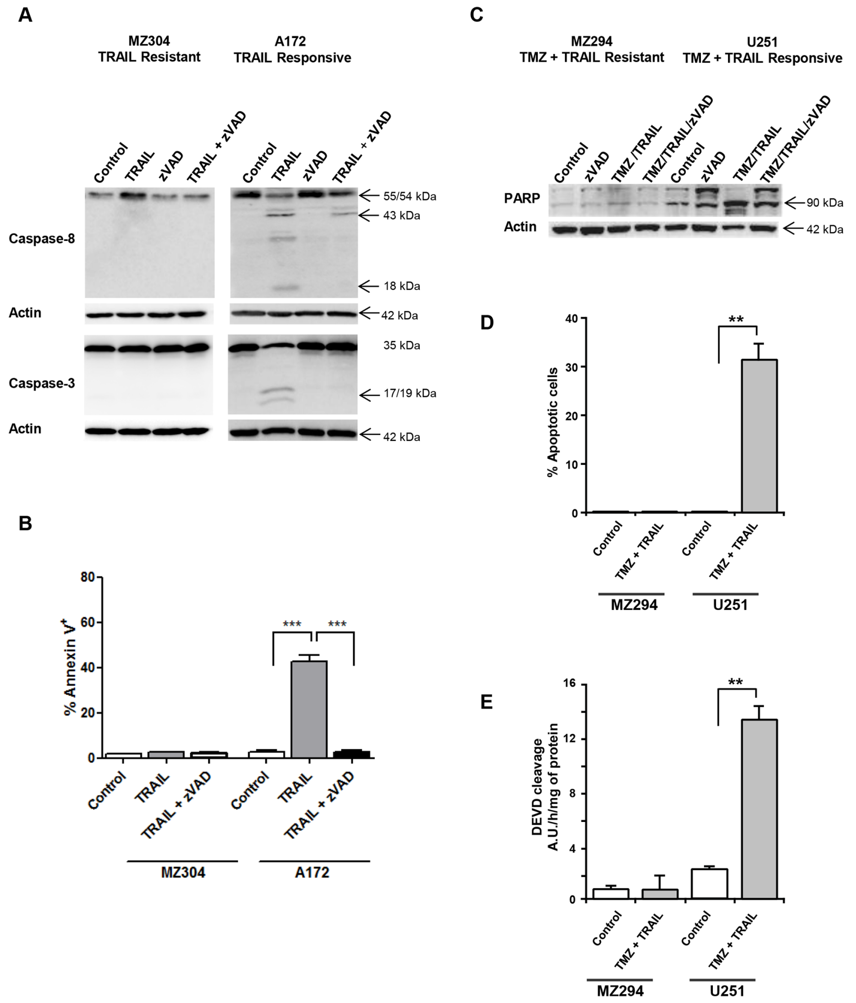

Supplementary Figure S1: Induction of extrinsic and intrinsic apoptotic pathways following treatment with TRAIL monotherapy and TRAIL and TMZ combined therapy. A. Cleavage of caspase-8 and caspase-3 was assessed in A172 and MZ304 cells following treatment with TRAIL (100 ng/ml) in the presence or absence of zVAD-fmk (150 µM) for 24 and 96 h, respectively. Cleavage of caspase-8 and caspase-3 was observed in the TRAIL-sensitive cell line, A172, but not in the TRAIL-resistant cell line, MZ304. In both cases, this cleavage was prevented by zVAD-fmk (n=3). **B.** Percentage of AnnexinV$^+$ A172 and MZ304 cells following treatment with TRAIL (100 ng/ml) in the presence or absence of zVAD-fmk (150 µM) for 96 h. The percentage of AnnexinV$^+$cells was significantly increased in the TRAIL-responsive cell line, A172, but not in the TRAIL-resistant cell line, MZ304. This increase in AnnexinV$^+$ A172 cells following TRAIL treatment was prevented by zVAD-fmk. Data are presented as mean ± SEM from n = 3 independent experiments. One-way ANOVA with post hoc Bonferroni test was performed for statistical analysis. **C.** Cleavage of PARP was assessed in U251 and MZ294 cells following the combined treatment of TRAIL (100 ng/ml) and TMZ (150 µM) in the presence or absence of zVAD-fmk (150 µM) for 48 h. Cleavage of PARP was observed in the TRAIL/TMZ-sensitive cell line, U251, but not in the TRAIL/TMZ-resistant cell line, MZ294. This cleavage was prevented by zVAD-fmk (n=3). **D.** Percentage of apoptotic cells following the combined treatment with TRAIL (100 ng/ml) and TMZ (150 µM) for 48 h. Apoptotic cell death, as assessed by nuclear fragmentation was significantly higher in the TRAIL/TMZ-sensitive cell line, U251, compared to untreated controls. No apoptotic cell death was detected in the TRAIL/TMZ-resistant cell line, MZ294. Data are presented as mean ± SEM from n = 3 independent experiments. Student's t test. **E.** Caspase-3 substrate cleavage was assessed in U251 and MZ294 cells following TRAIL (100 ng/ml) and TMZ (150 µM) treatment for 48 h. Caspase-3 substrate cleavage was significantly increased in the TRAIL/TMZ-sensitive cell line, U251, compared to untreated controls. No such increase was observed in the TRAIL/TMZ-resistant cell line, MZ294. Data are presented as mean ± SEM from n = 3 independent experiments. Student's t test. **p<0.01, ***p<0.001.

A

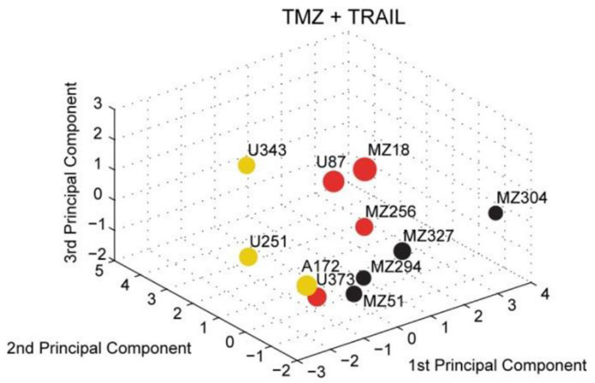

B

Drug	# Correctly Classified	% Correctly Classified
TMZ/TRAIL	10/11	91%

C

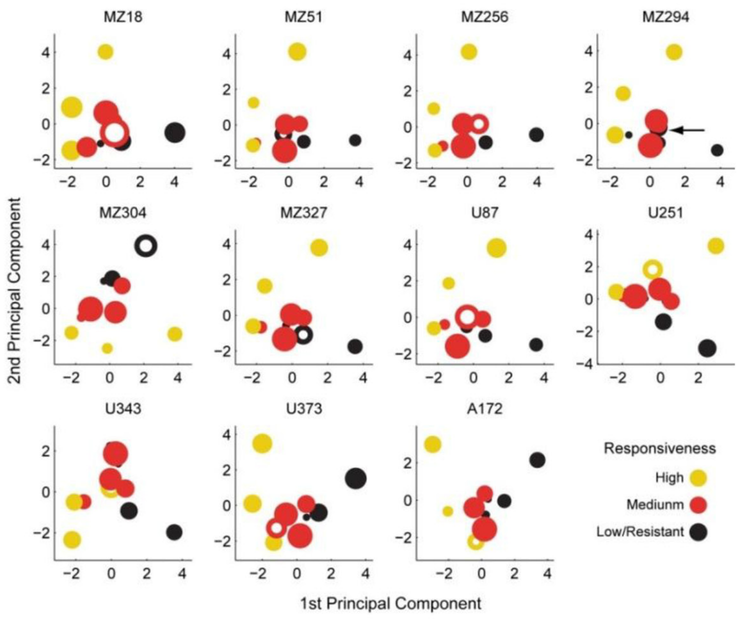

D

Drug	# Correctly Predicted	% Correctly Predicted
TMZ/TRAIL	6/11	55%

Supplementary Figure S2: Predicting responsiveness to TRAIL and TMZ combined therapy. A. Cell lines in the 3D PC space were color coded according to treatment responsiveness to TMZ and TRAIL combined treatment. Visual inspection indicates the response regions are spatially separated. **B.** Performance of the response group separation in the 4D PC space by linear discriminant analysis (LDA) is shown by listing the amount of correctly classified cell lines. **C.** 2D projections of the PC spaces calculated from combinations of 10 cell lines are shown for TMZ/TRAIL combined treatments. Circle sizes decrease with distance from the viewer, thereby providing information on the third PC dimension. Open circles represent the test cell lines, which were placed into the PC spaces according to their functional group values. **D.** Performance scores for correctly predicting cell line responses to TMZ/TRAIL combination therapy.

Supplementary Table S1: Protein expression amounts in the GBM cell line panel.

See Supplementary File 1

Supplementary Table S2: Functional group values

	BAK+BAX [uM]	BCL2+BCLXL +MCL1 [uM]	BID [uM]	BIM	Casp9* APAF1	CFLIP/ Casp8	(DR4+DR5) *FADD	PUMA	SMAC [uM]	XIAP/ Casp-3	Noxa
MZ18	2.429	0.582	0.22	6.02	6.9E-04	0.234	14.751	42	0.107	0.443	70.83
MZ51	3.183	0.528	0.086	7.9	7.8E-05	0.332	37.607	25.57	0.114	0.762	49.61
MZ256	2.188	0.628	0.163	40.18	9.6E-05	0.332	20.599	38.36	0.213	1.04	55.87
MZ294	2.989	0.45	0.092	3.8	2.8E-05	0.404	38.844	60.39	0.232	0.687	68.42
MZ304	2.047	0.419	0.069	48.63	3.0E-04	1.184	1.816	54.51	0.159	1.525	59.51
MZ327	1.768	0.452	0.129	12.28	9.5E-05	0.699	11.698	31.79	0.199	0.888	58.19
U87	2.771	0.502	0.227	13.21	3.6E-04	0.488	11.924	43.12	0.273	0.462	84.45
U251	2.096	0.447	0.116	98.43	7.5E-05	0.191	16.09	29.69	0.284	0.238	115.96
U343	4.45	0.82	0.088	153.53	1.4E-04	0.411	6.678	42.07	0.245	0.498	87.41
U373	2.002	0.411	0.079	20.54	3.1E-04	0.359	33.429	31.07	0.159	0.493	135.12
A172	2.268	0.237	0.163	27.16	2.6E-05	0.112	16.851	35.02	0.132	0.174	100.74

Note that as part of the subsequent PCA data are mean centered and normalised.

Supplementary Table S3: Weighting coefficients (Eigenvalues) of the FGs for the first four principle components

	PC1	PC2	PC3	PC4
BAK+BAX	0.00820605	0.47284539	0.00762253	0.45393557
BCL2+BCLXL+MCL1	0.13947871	0.4522717	0.22000561	0.37802581
BID	-0.1605908	-0.1337104	0.62081342	0.04106969
BIM	0.0041368	0.56225462	-0.031847	-0.2361781
Casp9*APAF1	0.0677543	-0.1457887	0.60829189	0.04696983
CFLIP/Casp8	0.54034879	-0.0619388	-0.0620612	-0.2604433
(DR4+DR5)*FADD	-0.2427457	-0.1844866	-0.3998817	0.46842554
PUMA	0.35595924	0.04948035	0.08728618	0.0181146
SMAC	-0.020966	0.39296198	-0.0243459	-0.3292395
XIAP/Casp3	0.55166499	-0.1169214	-0.1479703	0.01470614
Noxa	-0.4095096	0.09052452	-0.0351152	-0.4417802

Supplementary Table S4: Combination Index (CI) by Webb's Fractional Product Method for each cell line following TRAIL and Temozolomide treatment for 96 h

Cell line	Combination Index
MZ18	0.75592
MZ51	1.167037
MZ256	1.109937
MZ294	1.016221
MZ304	0.977444
MZ327	1.13935
U87	0.910931
U251	0.214254
U343	1.059717
U373	0.839497
A172	0.902034

CI < 1.0 indicates synergy; the lower the CI value is, the stronger the synergy
CI = 1.0 indicates additive effect
CI > 1.0 indicates antagonistic effect.
